# Modified polymeric biomaterials with antimicrobial and immunomodulating properties

**DOI:** 10.1038/s41598-024-58730-3

**Published:** 2024-04-05

**Authors:** Katarzyna Szałapata, Mateusz Pięt, Martyna Kasela, Marcin Grąz, Justyna Kapral-Piotrowska, Aleksandra Mordzińska-Rak, Elżbieta Samorek, Paulina Pieniądz, Jolanta Polak, Monika Osińska-Jaroszuk, Roman Paduch, Bożena Pawlikowska-Pawlęga, Anna Malm, Anna Jarosz-Wilkołazka

**Affiliations:** 1 Department of Biochemistry and Biotechnology, Institute of Biological Sciences, Maria Curie-Sklodowska University, Akademicka, 19, 20-033 Lublin, Poland; 2 Department of Virology and Immunology, Institute of Biological Sciences, Maria Curie-Sklodowska University, Akademicka 19, 20-033 Lublin, Poland; 3 Department of Pharmaceutical Microbiology, Medical University of Lublin, Chodzki 1, 20-093 Lublin, Poland; 4 Department of Functional Anatomy and Cytobiology, Institute of Biological Sciences, Maria Curie-Sklodowska University, Akademicka 19, 20-033 Lublin, Poland; 5 Present Address: Department of Biochemistry and Molecular Biology, Medical University of Lublin, Chodzki 1, 20-093 Lublin, Poland; 6 Present Address: Department of Pharmacology and Toxicology, National Veterinary Research Institute, Pulawy, Poland

**Keywords:** Biochemistry, Immunology, Microbiology

## Abstract

The modification of the surgical polypropylene mesh and the polytetrafluoroethylene vascular prosthesis with cecropin A (small peptide) and puromycin (aminonucleoside) yielded very stable preparations of modified biomaterials. The main emphasis was placed on analyses of their antimicrobial activity and potential immunomodulatory and non-cytotoxic properties towards the CCD841 CoTr model cell line. Cecropin A did not significantly affect the viability or proliferation of the CCD 841 CoTr cells, regardless of its soluble or immobilized form. In contrast, puromycin did not induce a significant decrease in the cell viability or proliferation in the immobilized form but significantly decreased cell viability and proliferation when administered in the soluble form. The covalent immobilization of these two molecules on the surface of biomaterials resulted in stable preparations that were able to inhibit the multiplication of *Staphylococcus aureus* and *S. epidermidis* strains. It was also found that the preparations induced the production of cytokines involved in antibacterial protection mechanisms and stimulated the immune response. The key regulator of this activity may be related to TLR4, a receptor recognizing bacterial LPS. In the present study, these factors were produced not only in the conditions of LPS stimulation but also in the absence of LPS, which indicates that cecropin A- and puromycin-modified biomaterials may upregulate pathways leading to humoral antibacterial immune response.

## Introduction

Biomaterials are used every day in several medical disciplines such as dentistry, orthopedics, urology, and several surgical specialties (neuro-, cardiovascular surgery etc.). Polymer-based biomaterials are used extensively in medical devices due to their advantageous properties, such as easy fabrication, inexpensiveness compared to metal materials, and biocompatibility. To decrease the risk of developing an infection, extensive work has been done on the functionalization of biomaterials to achieve their anti-infective behaviour^1^. These strategies can be categorized as passive systems (i.e. optimization of the biomaterial design and macro-/micro-architecture) or active strategies (combining antimicrobial therapeutics with biomaterials by physical or chemical modifications) ^2^.

Antimicrobial peptides (AMP) are an example of new substances with antimicrobial potential used in regenerative medicine as functional coatings, also as antibiofilm agents. These small molecules (on average 10–40 amino acids) are key elements of the innate immune system, and their antimicrobial activity is mainly related to such features as net positive charge, hydrophobicity, and flexibility. In addition, AMP molecules do not cause bacterial resistance and can thus be a real alternative to commonly used antibiotics^3^. Since AMP molecules are proteins, their potential use in regenerative medicine meets the latest trends based on coating biomaterials with various types of protein substances to increase cytocompatibility and improve their pro-adhesive properties^4^. Moreover, great emphasis is placed on the design of biomaterials that may exhibit immunomodulating properties of the human immune system. Major efforts are focused on the introduction of immuno-tolerant biomaterials in anti-inflammatory therapies^5^.

Given the above arguments, the next basic criterion that should be met by the biomaterial used in implantology is mechanical functionality. However, great attention should be paid to the reactions of the immune system to the presence of the implanted material. Thus, developing inflammatory reactions can cause the implant to fail in a process known as the foreign body response (FBR). This reaction is divided into two phases: an inflammatory response developing immediately after implantation and a repair phase involving the regeneration of damaged tissues. The phenomenon of distant reactions associated with FBR is quite common in cases of permanent implantation and is reflected by tissue fibrosis with a small amount of infiltrating cells or numerous infiltrations of mainly macrophages and lymphocytes. This phenomenon is therefore closely related to the developing inflammation that participates in the implant rejection reaction or leads to the limitation of its functionality^6,7^. One of the most important factors associated with the development of inflammation is cyclooxygenase-2 (COX-2). This enzyme participates in the synthesis of prostanoids, including prostaglandins, from arachidonic acid. Despite the undoubted advantages of the presence of this factor, its excess is associated with the development of pathological inflammation leading to tissue damage and rejection of implants. Moreover, the activation of innate mechanisms of the immune system, including macrophage infiltration, is a determinant of the activation of adaptive immunity, including humoral reactions. It includes not only the production of specific antibodies but also the release of growth factors, cytokines, or factors related to the development of inflammation ^8^. Mutual responses between implant-infiltrating immune cell populations are therefore associated with the release of a broad spectrum of pro- and anti-inflammatory cytokines. However, the scope and type of mediators are always related to the biomaterial used or its modification. Frequently, the responses to the implant include the release of IL-4 and IL-13 by pro-inflammatory Th2 lymphocytes or anti-inflammatory IL-10 produced by macrophages and B lymphocytes, which regulate the Th2 response^9^. It is therefore important to produce such implants that would be unattractive for adhesion by monocytes/macrophages and would stimulate the conversion of M1 pro-inflammatory macrophages into the M2 phenotype with anti-inflammatory activity recruiting cytokines such as IL-10 or TGF-β1^10^.

Every year, millions of patients undergo colorectal surgical procedures due to inflammatory bowel diseases and colorectal cancer. The resection and anastomosis of a part of the colon or rectum may lead to severe complications, starting with infections developing due to the surgery, through the risk of perioperative or postoperative death, and ending with decreased quality of life. Furthermore, in particular cases, anastomotic leakage may occur, and some patients may need re-operation^11–14^. Therefore, various approaches to regenerate colorectal tissue have been introduced^15,16^.

Proper tissue regeneration is related not only to the balanced activation of the immune system by the implant but also to the lack of toxicity to cells that, in normal conditions, are expected to colonize or come into contact with such biomaterial in the body. Biocompatibility describes the biological requirements of a biomaterial used in a medical device. More specifically, implanted material can function in the body with no local or systemic detrimental responses^17,18^. The biocompatibility of the material should therefore be understood as a property that prevents colonization by bacteria or fungi and, at the same time, coexists with the cells or tissues of the body without causing a toxic effect^19^. Therefore, these materials should be non-immunogenic. For this purpose, biomaterials either with a suitably modified structure or covered with an anti-adhesive agent are used. Generally, biomaterials must be non-toxic, easy to manufacture, and flexible, if required^6^.

The present study aimed to investigate extensively the antimicrobial potential of biomedical materials modified with two newly proposed antimicrobial substances (cecropin A and puromycin) and examine their potentially immunomodulatory and non-cytotoxic properties on the CCD 841 CoTr model cell line.

## Results

### Kinetics of the release of biologically active molecules from the surface of modified prostheses

**Figure 1:**
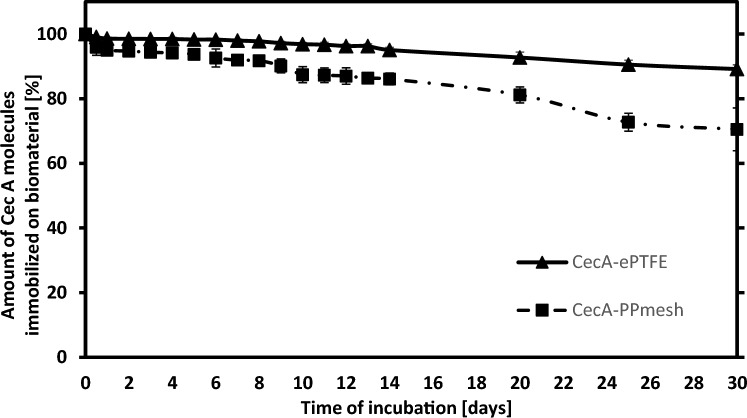
Kinetics of CecA release from modified surfaces of polymeric biomaterials (▲—CecA-ePTFE—vascular prosthesis modified with CecA, ■—CecA-PPmesh—surgical mesh prosthesis modified with CecA).

Based on the measurements, the amount of released Pur and CecA molecules was calculated during the 30-day incubation period. Pur was not released from the surface of the modified biomaterials in any of the analyzed samples. The data is presented in Fig. 1.

It was found that the newly formed covalent bond between Pur molecule and the surface of the two biomaterials was stable, and there was no release of the antibiotic from the surface of the modified polymers. In the case of the CecA-modified biomaterials, a slow release of antimicrobial peptide molecules was observed both from the surface of the PPmesh surgical mesh and the ePTFE vascular prosthesis. During the first two weeks of incubation, the small amount of CecA released was noted, while an intensification of the release of its molecules from the surface of the modified polymers was observed after this time. The total amount of released CecA particles from the surface of the modified biomaterials after 30 days was approx. 11% in the case of the CecA-ePTFE vascular prosthesis and approx. 30% in the case of the CecA-PPmesh surgical prosthesis.

### MIC and MBC analysis

The conducted experiments showed that both biologically active substances have antimicrobial potential against the reference microorganisms. The values presented in Table 1 indicate more effective antimicrobial action of CecA, in particular against gram-negative bacteria. In the case of *E. coli* and *P. aeruginosa* cells, an inhibitory effect was observed already at a concentration of 4 and 8 µg/mL, while the required concentration of CecA for gram-positive staphylococci was 30 µg/mL or 500 µg/mL. In the case of *C. albicans*, both the MIC and MBC values were 70 µg/mL. The Pur solution also exerted inhibitory and killing effects; however, the effective MIC values were 8 µg/mL for *E. coli*, 15 µg/mL for *S. aureus*, and 6 µg/mL for *S. epidermidis*. The highest Pur MIC and MBC values, i.e. 500 µg/mL, were obtained for *P. aeruginosa* and *C. albicans* strains (Table 1).

**Table 1:** MIC and MBC values (µg/mL) for CecA and Pur (tested range 1–500 µg/mL) relative to reference strains of *E. coli*, *P. aeruginosa, S. aureus, S. epidermidis*, and *C. albicans.*

Inhibitor	*E. coli* 0.83 × 10^2^ cfu/mL		*P. aeruginosa* 0.16 × 10^2^ cfu/mL		*S. aureus* 2.67 × 10^2^ cfu/mL		*S. epidermidis* 5.3 × 10^2^ cfu/mL		*C. albicans* 1.65 × 10^2^ cfu/mL	
	MIC	MBC	MIC	MBC	MIC	MBC	MIC	MBC	MIC	MBC
CecA	4	4	8	8	30	30	500	500	70	70
Pur	8	10	500	500	15	15	6	6	500	500

### Reduction of the growth of *S. epidermidis* in suspension cultures with modified PPmesh and ePTFE polymeric materials

In the case of the *S. epidermidis* suspension cultures, a decrease in density was observed after 18 h of incubation of the bacterial cells with the modified biomaterials (Table 2). A higher decline in the viability and density was caused by the action of the biomaterials modified with the use of the antimicrobial peptide (CecA). The culture density dropped to around 64% and 35% in the variants with the surgical mesh PPmesh and vascular prosthesis ePTFE, respectively.

**Table 2:** Reduction of *S. epidermidis* growth [%] in suspension cultures with unmodified and CecA- or Pur modified prosthesis.

Material	PPmesh	ePTFE
Unmodified materials	100.00 ± 7.31	100.00 ± 12.26
Pur-modified	75.95 ± 10.30	28.43 ± 7.28
CecA-modified	64.15 ± 4.95	35.46 ± 4.37

### SEM microphotographs of bacterial cultures on the surface of prostheses

**Figure 2:**
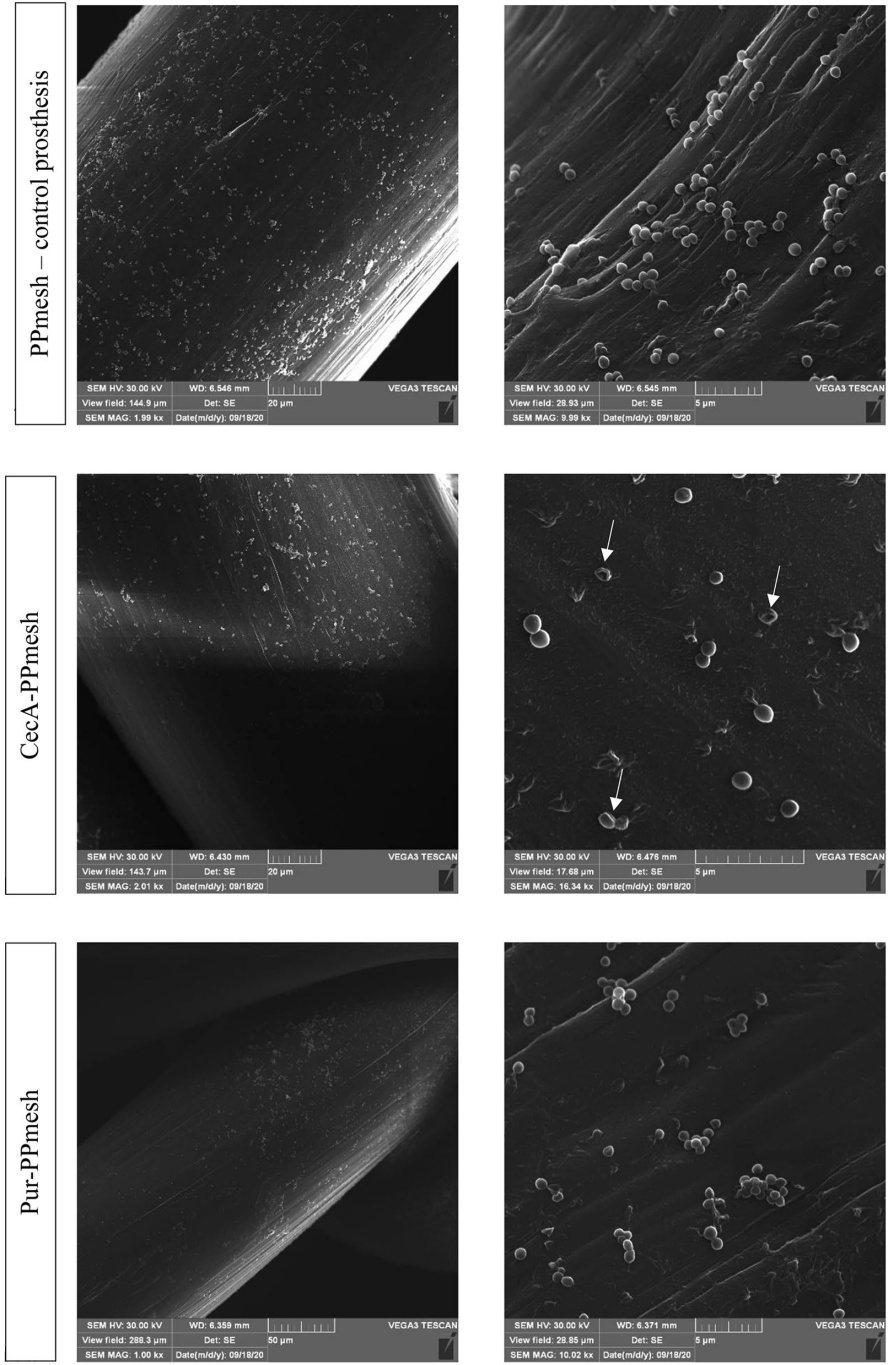
SEM microphotographs of PPmesh surgical mesh after incubation with *S. aureus* cells—control prosthesis (unmodified PPmesh), CecA-PPmesh, and Pur-PPmesh. White arrows mark cells with collapsed membrane as a result of the action of biologically active substances.

The analysis of the biofilm formation on the surface of polymer prostheses showed that the PPmesh modification with the use of CecA resulted in a decrease in the viability of microorganisms living on its surface. This was evidenced by the slower conversion of TTC to red formazan, which indirectly proves the metabolic activity of the tested microorganisms. Based on these results (Supplementary materials—Table S1 and Table S2), the best samples were selected and SEM microphotographs were taken. Then, changes in the microbial culture density and the morphology of the microbial cells were observed (Fig. 2). In the CecA-modification variant, a decrease in the number of microorganisms on the surface of the biomaterial and characteristic indentations in the cell membrane caused by the action of the antimicrobial peptide were observed.

### Antimicrobial potential against clinical gram-negative strains

The MIC and MBC analysis of the activity of CecA against selected clinical isolates of gram-negative bacteria showed that the determined inhibitory concentrations resulted in bacterial cell death in both *E. coli* and *P. aeruginosa* (Table 3). In the case of most clinical isolates, the values of the inhibitory and bactericidal concentrations were similar to those determined for the reference strains. The *P. aeruginosa* P9, P11, and P21 isolates required the highest effective concentrations of CecA—16 µg/mL.

**Table 3:** MIC and MBC values (µg/mL) for CecA in the case of *E. coli* and *P. aeruginosa* clinical strains isolated from skin and soft tissue infections; bacterial suspension density: 1 × 10^2^ cfu/mL.

*E. coli*			*P. aeruginosa*		
Strain No	MIC	MBC	Strain No	MIC	MBC
E8	4	4	P1	4	4
E9	2	2	P2	8	8
E10	4	4	P3	8	8
E14	4	4	P4	8	8
E25	4	4	P5	8	8
E26	4	4	P6	8	8
E27	4	4	P7	8	8
E28	4	4	P8	4	4
E29	4	4	P9	16	16
E30	4	4	P10	8	8
E58	4	4	P11	16	16
E61	4	4	P12	8	8
E62	2	2	P13	8	8
E63	4	4	P14	8	8
E70	2	2	P15	8	8
E72	2	2	P16	8	8
E77	2	2	P17	8	8
E78	2	2	P18	4	4
E80	2	2	P20	8	8
E82	2	2	P21	16	16
ATCC 25922	4	4	ATCC 27853	8	8

### Reduction of the growth of clinical gram-negative strains in the presence of unmodified and CecA-modified PPmesh

The analysis of the growth reduction in the suspension cultures did not show any inhibitory effect in the variants with the unmodified and CecA-modified PPmesh prostheses on the growth of the reference *E. coli* and *P. aeruginosa* strains and their clinical isolates (Table 4).

**Table 4:** Density of gram-negative bacteria suspension cultures (*E. coli* and *P. aeruginosa*) in contact with the unmodified and CecA-PPmesh—data presented as a mean log10 (log_10_ cfu/mL). The experiment was carried out in 3 replications.

Strain	Initial density of bacterial suspension	after 24 h of incubation	
		unmodified PPmesh	CecA-PPmesh
*E. coli*			
E9	6.2	10.23	10.23
E62	6.0	10.37	9.93
E70	5.97	10.27	9.8
E72	6.1	10.37	9.83
E77	6.1	10.37	10.07
E78	6.0	10.43	9.73
E80	6.03	10.63	9.83
E82	6.17	10.57	9.97
ATCC 25,922	5.77	9.97	10.7
*P. aeruginosa*			
P1	5.8	10.37	10.6
P8	5.73	10.9	10.53
P9	6.0	11.3	10.9
P11	5.9	11.17	10.53
P18	5.83	10.93	10.8
P21	5.87	10.2	10.37
PA ATCC 27853	5.9	10.63	10.63

### Effect of unmodified and modified PPmesh prostheses and their extracts on cell proliferation and cytotoxicity

#### Effect of soluble CecA and Pur on CCD 841 CoTr cells

**Figure 3:**
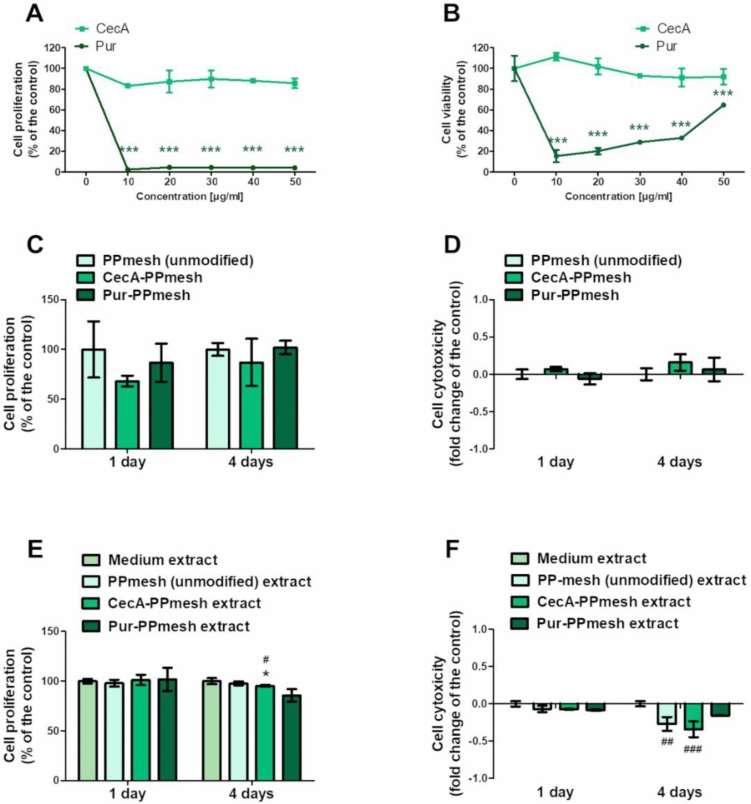
Effect of CecA and Pur on the cell proliferation and cytotoxicity in 2D cultures (**A**–**B**) and 3D cultures: direct (**C**–**D**) and indirect (**E**–**F**) tests, measured with MTT (proliferation) and LDH (cytotoxicity) methods; statistically significant results compared to the control are indicated with ‘*’ and those compared to the medium are marked with “^#^”: *—*p* < 0.05, **—*p* < 0.01, ***—*p* < 0.001 (One-Way ANOVA, Dunnett’s test). control – PPmesh (unmodified), commercial prosthesis; CecA-PPmesh—prosthesis modified with cecropin A; Pur-PPmesh—prosthesis modified with puromycin; medium—extract prepared without the prosthesis.

MTT and LDH assays were performed to evaluate the effect of soluble CecA and Pur on the cells. CecA did not exert any harmful effect on CCD 841 CoTr cells (the strongest effect was exhibited at 50 µg/mL, with a decrease in proliferation to 79.5 ± 9.7% and a decrease in viability to 91.9 ± 5.4% of the control; however, the results were not statistically significant) (Fig. 3A–B). Pur, on the other hand, exhibited a strong viability and proliferation-limiting effect. Proliferation was reduced almost completely at all concentrations, while the viability was affected most potently by the lower concentrations, with a dose-dependent decrease (the viability of the cells was 15.4 ± 4.9% at 10 µg/mL and 64.7 ± 1.4% of the control at 50 µg/mL) (Fig. 3A–B).

#### Effect of the CecA and Pur modification of PPmesh on CCD 841 CoTr cells

The CecA- and Pur-modified prostheses did not exhibit significant proliferation-limiting or cytotoxic effects. In the direct test, CecA caused a slight decrease in proliferation; however, the result was not statistically significant (Fig. 3C, D). Interestingly, the effect was stronger after 1 day than after 4 days (a decrease to 68.1 ± 5.4% vs. 86.9 ± 23.8% of the control). Furthermore, a statistically significant lower LDH level was observed in the case of the CecA- and Pur-modified prosthesis extracts, compared to the unmodified prosthesis (control) and medium extracts (Fig. 3E, F).

### Inflammatory response of cells cultured on the prostheses

#### NO secretion

**Figure 4:**
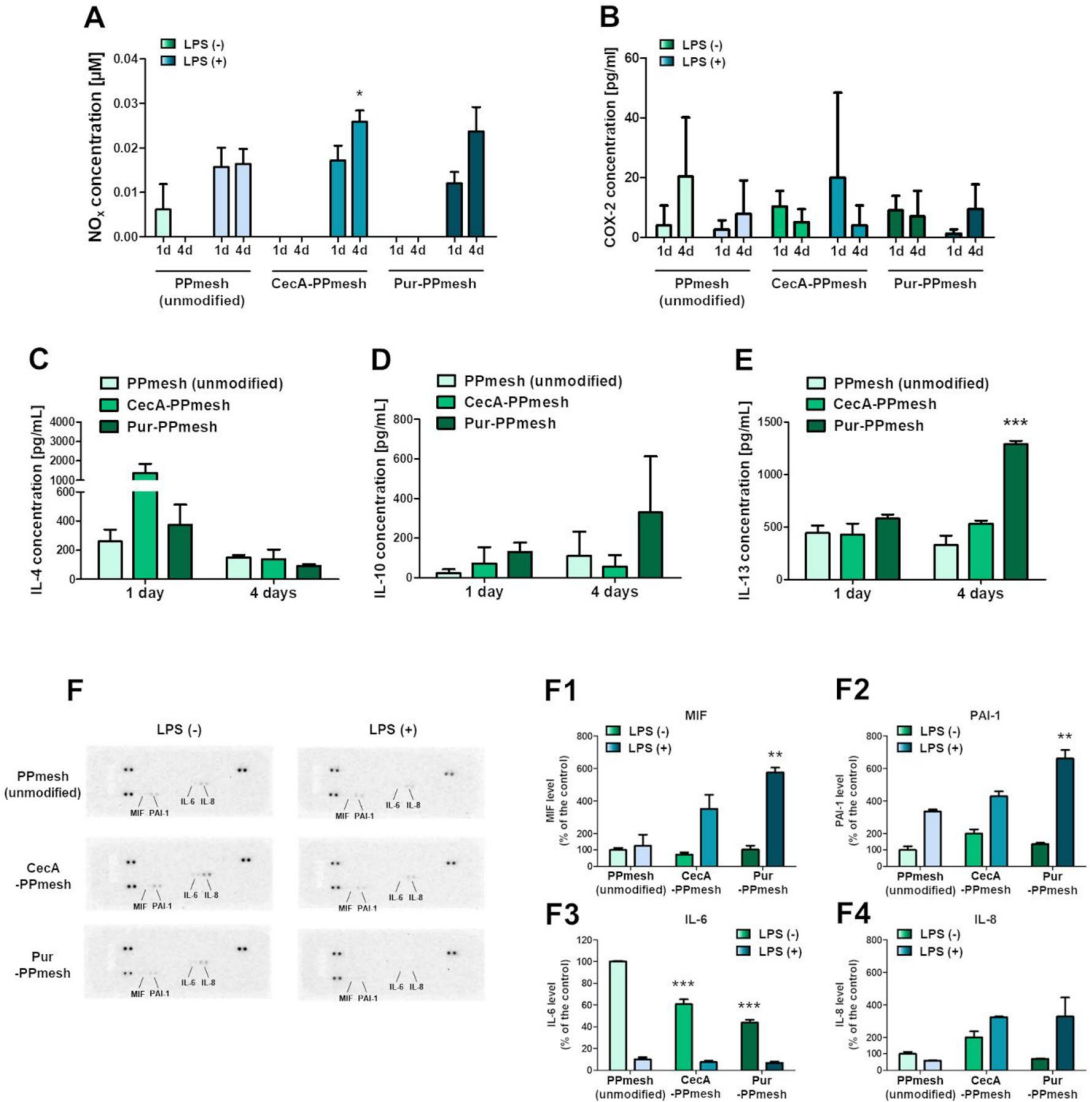
Effect of the presence of CecA and Pur on the surface of PPmesh on the cell inflammatory response: NO secretion (Griess method) (**A**), COX-2 concentration (ELISA) (**B**), Th2-type immunological response cytokines (ELISA): IL-4 (**C**), IL-10 (**D**), and IL-13 (**E**), and inflammatory mediators panel (cytokine array) (**F**): MIF (F1), PAI-1 (F2), IL-6 (F3), and IL-8 (F4) levels; statistically significant results are indicated with ‘*’: *—*p* < 0.05, **—*p* < 0.01, ***—*p* < 0.001 (One-Way ANOVA, Tukey’s test). Control—PPmesh (unmodified), commercial prosthesis; CecA-PPmesh—prosthesis modified with cecropin A; Pur-PPmesh—prosthesis modified with puromycin; 1d/4d—cells cultured on the prostheses for 1 or 4 days; LPS(+) indicates cultures pre-incubated with 10 µg/ml LPS for 2 h.

Nitric oxide was secreted only by the cells pre-incubated with 10 µg/mL LPS (excluding the control after 1 day; however, the concentration was very low—0.006 ± 0.0057 µM). The level of NO_x_ secreted by the LPS-treated cells cultured on the CecA- and Pur-modified PPmesh prostheses was similar to the control after 1 day (0.016 ± 0.0044, 0.017 ± 0.0033, and 0.012 ± 0.0025 µM secreted by cells cultured on the control prostheses, CecA-, and Pur-modified PPmesh prostheses, respectively) and higher after 4 days (0.016 ± 0.0033, 0.026 ± 0.0025, and 0.024 ± 0.0055 µM secreted by cells cultured on the control prostheses, CecA-, and Pur-PPmesh prostheses, respectively) (Fig. 4A). The results may indicate pro-inflammatory properties of the CecA- and Pur-modified prostheses; therefore, further analyses were performed.

#### COX-2 concentration

The analysis of the COX-2 concentration in the cells cultured on the prostheses revealed interesting results. In the case of the control prostheses, the COX-2 level increased with time, while the COX-2 concentration in the CecA and Pur modification variants was lower after 4 days of incubation compared to the 1st day of incubation. The phenomenon was observed in both the absence and presence of LPS incubation, excluding Pur LPS(+). The concentration of COX-2 increased 5.2-fold and 3.0-fold (in the absence and presence of LPS, respectively) in the cells cultured on the control prosthesis and dropped 2.0-fold and 5.1-fold in the cells on the CecA-modified prosthesis (in the absence and presence of LPS, respectively) and 1.3-fold in the cells on the Pur-modified prosthesis (in the absence of LPS) (Fig. 4B).

#### IL-4, IL-10, and IL-13 concentration

One of the aspects of the study was to assess the antimicrobial activity of the modified prostheses. Therefore, the concentration of IL-4, IL-10, and IL-13, i.e. cytokines engaged in activation and development of humoral (Th2-type) immunity, was evaluated.

The level of IL-4 secreted after 1 day by the cells on the CecA- and Pur-PPmesh prostheses was higher than the amount released by the cells on the control prostheses (unmodified): 258.9 (PPmesh), 1366.3 (CecA-PPmesh), and 372.2 pg/mL (Pur-PPmesh). However, with time, the concentration of secreted IL-4 decreased to 149.8 (unmodified), 136.4 (CecA-modified), and 90.1 pg/mL (Pur-modified) after 4 days of incubation (Fig. 4C).

The production of IL-10 by the cells on the modified prostheses was higher than in the control after 1 day: 22.0 (unmodified), 72.5 (CecA-modified), 130.0 (Pur-modified) pg/mL. After 4 days, the concentration of secreted IL-10 increased in the case of the unmodified and Pur-modified prostheses (to 109.6 and 329.9 pg/mL) and slightly decreased in the case of the CecA-modified prosthesis (55.9 pg/mL) (Fig. 4D).

The concentration of IL-13 secreted by the cells after 1 day was similar in the control prostheses (444.9 pg/mL) and in the case of the CecA-modified prosthesis (427.6 pg/mL), and higher in the case of Pur-modified prosthesis (580.8 pg/mL). After another 3 days, the level of IL-13 secreted by the cells on the unmodified prosthesis decreased (to 331.1 pg/mL) but increased in the case of cells on the CecA- and Pur-modified prostheses (to 533.1 and 1288.1 pg/mL, respectively) (Fig. 4E).

It appears that the Pur-modified and CecA-modified prostheses had the strongest potential to modulate Th2-type immunity through IL-13, while CecA-modified prosthesis – through IL-4. At the same time, the modified prostheses had a distinct effect on the level of anti-inflammatory IL-10, i.e. the CecA-modified prosthesis appeared to decrease its secretion by the cells, while the Pur-modified prosthesis increase the release.

#### Inflammatory panel

Previous analyses revealed that the COX-2 mediator of inflammation was induced during growth of the cells on the prostheses. Furthermore, the secretion of Th2-type immunity-related cytokines, IL-4, IL-10, and IL-13, was modulated. Therefore, the proteome profiler for cytokines was used to detect other cytokines that may be secreted by the cells. The signals from four cytokines, i.e. MIF, PAI-1, IL-6, and IL-8, were sufficiently strong for measurement.

The level of MIF secreted by the cells on the CecA- and Pur-modified prostheses (72.6 ± 12.2 and 102.7 ± 34.2% of the control—unmodified prostheses) (Fig. 4F1) was similar to the amount secreted by the cells on the unmodified prosthesis in the LPS(−) conditions. After the LPS pre-incubation, increased MIF secretion was noted in the samples with all the prosthesis variants; however, the increase was much stronger in the case of the CecA- and Pur-modified prostheses (a 4.9 and 5.6-fold increase compared to the 0.25-fold increase in the control) (Fig. 4F1).

The level of PAI-1 secretion was higher in the case of cells on the CecA- and Pur-modified prostheses than in the unmodified variant in both LPS(−) and LPS(+) conditions. The highest amount in the absence of LPS was secreted by the cells on the CecA-modified prosthesis (201.5 ± 35.5% of the control) and the highest amount in the presence of LPS was secreted by the cells on the Pur-modified prosthesis (662.0 ± 73.1% compared to the control – unmodified prostheses). The LPS pre-incubation induced the production of PAI-1 about 3.4-fold (unmodified), 2.1-fold (CecA-modified), and 4.8-fold (Pur-modified) (Fig. 4F2).

The level of IL-6 secreted by the cells cultured on the modified prostheses was much lower than in the case of the unmodified prosthesis in both LPS(−) and LPS(+) conditions. Furthermore, the pre-incubation with LPS caused a decrease in IL-6 secretion by the cells on all the prostheses (10, 8.1, and 6.3-fold in the case of the cells grown on the unmodified, CecA- and Pur-modified prostheses, respectively) (Fig. 4F3).

IL-8 secretion in the LPS(−) conditions was induced in the cells on the CecA-modified prosthesis and decreased in the cells on the Pur-modified prosthesis, compared to the unmodified biomaterials (to 202.3 ± 51.0 and 70.6 ± 3.1%, respectively). After the pre-incubation with LPS, the level of the secreted IL-8 was 1.7-fold lower in the case of the cells on the unmodified prosthesis and 1.6-fold and 4.7-fold higher in the samples of the cells on the CecA- and Pur-modified prostheses, respectively (Fig. 4F4).

## Discussion

The growing problem of antibiotic resistance among many strains of nosocomial pathogens means that emphasis is increasingly being placed on the design of release-active and antimicrobial biomaterials. According to WHO statistics, it is estimated that 10 million patients may die annually by 2050 as a result of infections with multidrug-resistant strains of pathogens^20^. The six resistant strains that most commonly cause infections include *Escherichia coli, Staphylococcus aureus, Klebsiella pneumoniae, Streptococcus pneumoniae, Acinetobacter baumannii*, and *Pseudomonas aeruginosa*^21^. In the case of surgical meshes, infections affect about 1–4% of all patients, but on a global scale there are tens of thousands of people a year who are subjected to difficult therapy^22^. Therefore, from the WHO point of view, it is important to actively monitor and introduce new, safe antimicrobial agents^23^ and new methods that limit the colonization of biomaterials by bacteria and the production of biofilm by these microorganisms.

Surgical Site Infections (SSIs) are a common complication in the case of surgeries, including operations on the colon^11,12,24,25^. Development of biomaterials to regenerate the resected part of the colon with antibacterial properties would be beneficial for millions of patients. Furthermore, the limitation of the antimicrobial drug release in the site of the intervention could contribute to a reduced use of systemic antibiotic therapy.

The introduction of new biomaterial modification techniques is possible e.g. thanks to the use of AMP. By 2016, approximately 3000 AMP molecules with potential biomedical applications had been identified and characterized, but most of them are not possible to use in human therapy in their natural form^26^. To increase the stability and safety of AMP use, several immobilization techniques on biomaterials are introduced using physical and chemical methods (based, for example, on the formation of a covalent bond)^27^. In addition to the glutaraldehyde cross-linking agent, which ensures high durability and stability of molecules immobilized on the surface of biomaterials, other methods are also used to increase the stability and biocompatibility of antimicrobial peptides. An example is the p(HEMA) hydrogel, which can actively release antimicrobial molecules—ampicillin trihydrate and levofloxacin thanks to its sensitivity to pH changes^28^. On the other hand, experiments are conducted on self-assembled peptides based on ovalbumin-derived peptide TK913, which show increased stability to the action of trypsin^29^. Additionally, structures of bio-inspired "viral" liposomes are created; they contain AMP molecules “spikes” on their surface and ensure better-controlled penetration and increased stability of biomolecules, causing a bactericidal effect and removing bacterial biofilm^30^.

Immobilization of CecA and Pur with glutaraldehyde yielded very stable preparations of modified biomaterials, which released small amounts of biomolecules from their surface even after 30 days of incubation (in the case of CecA, 11–30% of the immobilized molecule was released). In the case of electrospun membranes loaded with atorvastatin, approximately 90% of the substance was released from the mesh surface after 10 days from the start of incubation, thus supporting the growth of HUVEC cells and the angiogenesis process^31^. Approximately 60% of the PEP-1 molecule used in composite meshes was released from the mesh surface within 10 days, and it did not exert a cytotoxic effect on HDF cells^32^.

The highest efficiency of CecA was observed against strains of gram-negative bacteria. The results obtained for the reference strains were also confirmed for the clinical strains, for which the MIC values ranged from 2–4 µg/mL for *E. coli* and 4–16 µg/mL for *P. aeruginosa*. Tests performed on the dadapin-1 peptide showed that the MIC values were in the range of 3.1–6.2 µM for gram-positive bacteria *S. aureus*, *S. epidermidis*, and *S. warneri* and higher (12.4–24.9 µM) for gram-negative bacteria *E. coli* and *P. aeruginosa*, respectively^33^. On the other hand, the hybrid LfcinB6 and KR-12-a4 peptide showed MIC values of 4–8 µM against 12 bacterial strains, including MRSA, *S. aureus*, *B. subtilis*, and *P. aeruginosa*^34^. In turn, the modified TICbf-14 peptide reduced the swimming motility of a *P. aeruginosa* strain and increased the stability to degradation by trypsin digestion^35^. Interestingly, Peng et al.^36^ showed a highly effective fungicidal effect of cecropin against the *C. albicans* CMCC(F)98,001 strain at values of 1.8 µg/mL, while our research on the *C. albicans* ATCC 10231 strain showed the fungicidal activity of CecA at 70 µg/mL.

Unfortunately, despite the promising effect of CecA in the native form on the reference strains and the confirmation of the inhibition of the production of *S. aureus* biofilm on the biomaterial surface, as well as the reduction of the number of *S. epidermidis* cells in the suspension cultures in contact with the modified biomaterial, no growth reduction of the clinical strains of *E. coli* and *P. aeruginosa* was observed. This phenomenon may be related to the conformational rigidity and the relatively short linker arm provided by glutaraldehyde. The increase in the conformational freedom had a positive effect in the case of tachyplesin I tagged with the polyhydroxyalkanoate-granule-associated protein and immobilized on poly(3-hydroxybutyrate-co-3-hydroxyvalerate) (PHBHV) via hydrophobic interaction. The polymer modified in this way showed inhibition of the growth of gram-positive (*S. aureus, B. cereus*) and gram-negative bacteria (*E. coli, P. aeruginosa*)^37^. Inhibition of biomaterial colonization was also demonstrated using LL37, magainin 2, and parasin I on plasma polymer interlayer platforms, even at high bacterial culture densities of 1 × 10^7^ cfu/mL^38^. Another example with increased stability than in its native form is the RRP9W4N peptide covalently attached to amphiphilic and ordered mesoporous Pluronic F127 hydrogels, which showed high antimicrobial activity against *S. epidermidis, S. aureus, P. aeruginosa*, methicillin-resistant *S. aureus* (MRSA), and multi-drug resistant *E. coli* for up to 24 h of action^39^.

Polypropylene has been used as a biomaterial for cell growth for years. It is used in medicine for numerous purposes, especially such surgery as hernia treatment or vaginal reconstruction. PP is a relatively cheap and durable material facilitating cell adhesion and growth^40,41^. The study aimed to determine whether the immobilization of two antimicrobial agents, cecropin A and puromycin, on the PP mesh may enhance the antibacterial effect of prostheses without a simultaneous harmful effect on endothelial cells.

CecA did not exert any significant effect on the viability or proliferation of the CCD 841 CoTr cells, regardless of its soluble or immobilized form. It has been demonstrated that, unless administered at high concentrations (i.e. over 40 µM ≈ 160 µg/ml), CecA does not exert a cytotoxic effect on either cancer or non-cancer cells, e.g. MDA-MB-31 (breast cancer) and M14K (pleural mesothelioma)^42^, K562 (chronic myelogenous leukaemia), U937 (histiocytic lymphoma), THP-1 (acute monocytic leukaemia), HEK-293 (normal embryonal kidney cells), and PBMCs (primary peripheral blood mononuclear cells)^43^, and NIH 3T3 (embryonal fibroblasts)^44^.

In contrast, Pur did not induce a significant decrease in the cell viability or proliferation in the immobilized form (Pur-PPMesh) but significantly decreased cell viability and proliferation when administered in the soluble form. Its cytotoxic and pro-apoptotic effects towards mammalian cells have been demonstrated before^45^. The phenomenon of cytotoxicity of the soluble form of Pur, and not the immobilized form, may be related to its mechanism of action—the agent is responsible for the elongation termination during translation due to the A-site of the ribosome binding and the prevention of the aa-tRNA binding, leading to the release of the peptide and disassembly of the ribosome. Furthermore, the compound may lead to disruption of the plasma membrane^46^. It has been demonstrated that Pur may exhibit cytotoxicity towards cells already at a low concentration, as it has been reported to induce a significant viability decrease in colorectal cancer cells HCT116, SW620, and H1299 at 0.25 µg/ml^47^ and a complete viability decrease in HepG2 (hepatocellular carcinoma) and primary rat hepatocytes at 10 µM ≈ 5.4 µg/ml^48^.

Inflammation is a process of preparation and response of the organism to harmful stimuli, such as infection. It leads to the recruitment of immunocompetent cells to the site; immunological cells are suited to recognize and eliminate factors that may pose a threat to the organism. However, the inflammation and immune response at the site of the transplant may lead to its rejection; furthermore, it has been demonstrated that implantation of the biomaterial into the organism may lead to chronic inflammation, tissue damage, and implant rejection in the course of foreign-body reaction if the process is not managed^49–52^. Therefore, we have studied the response of CCD 841 CoTr cells cultured on the prostheses regarding the production of particular inflammation- and immune response-related factors. The idea was to examine the potential of the cells cultured on the prostheses to immunomodulate the microenvironment. The best effect would be the polarization of the response so that chronic inflammation and immune response against cells would be prevented with the antibacterial effect boosted simultaneously.

COX-2 is one of the main mediators of inflammation and one of the factors responsible for the development of chronic inflammation. We demonstrated that the level of COX-2 in the cells changed depending on the time of growth on the PPmesh prostheses and the presence or absence of LPS. The most promising results were exhibited by the CecA-PPmesh—the concentration of COX-2 was higher in the LPS pre-incubated cells, which indicates the proper response to the inflammation and dropped with time, with lower levels after 4 days than after 1 day. This may suggest that cells cultured on the biomaterial modified with CecA tend to react properly to the infection and, at the same time, show no likelihood of developing chronic inflammation. Similar results were obtained in the case of IL-4 and IL-10, i.e. the concentration of the cytokines secreted by the cells cultured on the CecA-PPmesh was initially higher but declined with time, suggesting the proper suppression of the immune response. Epithelial cells, including those of intestine origin, are capable of secreting a number of pro- and anti-inflammatory cytokines, including IL-4, IL-6, IL-10, and IL-13, which participate in the humoral response, mainly through mediating the switch to the production of IgG and IgM, highly responsive in the case of bacterial infections^53–57^. Furthermore, IL-4 may inhibit the production of IL-2 and IFN-γ specific for Th1-type cellular-dependent immune response^56^. IL-6 mediates a high number of immunological processes, including the formation of a link between innate and adaptive immunity or the stimulation of B cells to differentiate into plasma cells and produce antibodies (mainly IgG4); however, IL-6 may be engaged in several pathological processes and diseases, including chronic inflammation. We demonstrated that the levels of IL-6 produced by cells cultured on the CecA- and Pur-PPmesh were higher than those produced by cells grown on the unmodified PPmesh. This may indicate that the modification of the prostheses with CecA and Pur could limit the possibility of biomaterial-induced chronic inflammation. Similar results were demonstrated by Xu et al., where cecropin B-coated titanium prostheses caused lower production of TNF-α and IL-6 by macrophages (RAW 264.7 cell line) than uncoated ones, and by Wang et al. where cecropin B and cecropin DH decreased the production of TNF-α and IL-6 by LPS-stimulated RAW264.7 cells^58,59^. Polypropylene meshes were demonstrated by Di Vita et al. to induce the production of factors mediating inflammation (CRP, IL-1, IL-1Rα, IL-6, IL-10, α1-AT) in patients with hernia. Initially, the levels of IL-1, IL-6, IL-10, and IL-1Rα were higher but decreased with time^60^. This may suggest that the implantation of the polypropylene mesh into tissues induces an immune response, but the reaction is acute rather than chronic. Similar results were demonstrated in the present study. Another detected cytokine was IL-8 (CXCL-8), i.e. an interleukin/chemokine responsible for the chemoattraction of leukocytes, mainly neutrophils. It also mediates trafficking and degranulation of neutrophils and stimulates phagocytosis^61,62^. Its increased level may lead to neutrophil-dependent antibacterial immunity. Another noteworthy cytokine whose production was increased is the macrophage migration inhibition factor (MIF). The cytokine mediates mainly innate immunity but may also play an important role in adaptive immunity. MIF upregulates several factors, including COX-2, NO, IL-6, and IL-8. Importantly, its mechanism is based on, among other things, interaction with TLR4^63–65^. Finally, we demonstrated that plasminogen activator inhibitor-1 (PAI-1) was induced in the CecA- and Pur-PPmeshes case. Its main role is to inhibit tissue and urokinase plasminogen activators; however, its role in immunity has been demonstrated by a number of studies^66^. For example, the lack of PAI-1 may lead to a more severe outcome of bacterial infection, e.g. with *Streptococcus pneumoniae*, *Haemophilus influenzae* or *Yersinia enterocolitica*^67–69^. Therefore, the increased production of PAI-1 by the cells grown on the studied prostheses may mediate their antibacterial properties.

The CecA-PPmesh and, to some extent, the Pur-PPmesh were demonstrated to mediate a number of immune factors, mainly COX-2, IL-4, IL-6, IL-10, IL-13, and MIF. Possibly, the key regulator of this activity may be related to TLR4, a receptor recognizing bacterial LPS^54,70^. Indeed, various cecropins have been demonstrated to induce TLR4^71,72^. In the present study, these factors were produced not only in the conditions of LPS-stimulation but also in the absence of LPS. This indicates that the CecA- and Pur-modified PPmesh may upregulate pathways leading to humoral antibacterial immune response.

The CecA- and Pur-PPmesh materials exhibited significant antibacterial activity. The experiments on CCD 841 CoTr cells revealed that, in addition to the antimicrobial properties of the agents alone, they may also induce the production of antibacterial and immune response-stimulating cytokines, such as IL-4, IL-13, MIF, and IL-8.

## Conclusions

The development of antimicrobial coatings for different polymeric substrates is crucial for reducing bacterial adhesion and proliferation on biomaterials and biofilm formation, which is the primary cause of bacterial infections associated with biomaterials. The modification of the surgical polypropylene mesh and the polytetrafluoroethylene vascular prosthesis with the tested active substances yielded very stable biomaterials exhibiting significant antibacterial activity. The results proved that this modification also resulted in the production of antibacterial and immune response-stimulating cytokines. The key regulator of this immune activity may be related to TLR4, a receptor recognizing bacterial LPS, which was produced not only in the conditions of LPS stimulation but also in the absence of LPS. The modification of the surgical propylene mesh may upregulate pathways, leading to a humoral antibacterial immune response. This may suggest that cells cultured on the modified biomaterial tend to react appropriately to the infection and, at the same time, show no likelihood of developing chronic inflammation. The immobilization of the active substance on the biomaterial surface affects the activity of the substance on the one hand and, on the other hand, imparts new properties to the modified surface. The activity of immobilized and non-immobilized molecules varies, which should be considered in tests of the antimicrobial activity of various substances. What is more, the cytotoxicity of the tested substances may change (increase or decrease) after their immobilization on the surface of the biomaterial.

## Materials and methods

To confirm the hypothesis, the following experiments were proposed: determination of the kinetics of the release of biologically active molecules to check the durability of their attachment to the biomaterial, MIC and MBC analysis for reference strains and clinical isolates from skin and soft tissue infections, analysis of biofilm formation on the surface of biomaterials combined with SEM imaging, evaluation of the reduction of the growth of pathogenic microorganisms in suspension cultures, evaluation of the cytotoxicity of modified biomaterials and their influence on CCD 841 CoTr cell line proliferation, and a whole panel of immunomodulatory analyses using the Griess method, ELISA tests, and cytokine array.

### Chemicals

Chemical reagents used to modify the surface structure of the biomaterials were purchased from Sigma Aldrich (puromycin—Pur, glutaraldehyde—GLA) and from ABCR, Gute Chemie (cecropin A—CecA). Different culture media were used in the different experiments: Mueller–Hinton Bullion (MHB, Sigma Aldrich) for the MIC/MBC analysis and the reduction of bacterial growth in liquid broth, Yeast Malt Broth (YMB, Sigma Aldrich) for the reduction of yeast growth in liquid broth and the yeast biofilm assay, and Tryptic Soy Broth (TSB, Sigma Aldrich) for the bacterial biofilm assay.

### Microorganisms

The following reference microorganism strains were used in the antimicrobial evaluation studies: *Escherichia coli* ATCC® 25922™, *Pseudomonas aeruginosa* ATCC® 27853™, *Staphylococcus aureus* ATCC® 25923™, *Staphylococcus epidermidis* ATCC® 14990™, and *Candida albicans* ATCC® 10231™ (American Type Culture Collection, USA). The antimicrobial potential was also tested for selected clinical isolates of *Escherichia coli* and *Pseudomonas aeruginosa* obtained from skin and soft tissue infections (the deposit of the Medical University of Lublin, Department of Pharmaceutical Microbiology).

### Polymeric biomaterials

The modification of the surface structure of the polymeric biomaterials was carried out on PPmesh—surgical polypropylene, non-absorbable mesh (Premilene Mesh, B|Braun, Spain) and ePTFE—polytetrafluoroethylene vascular prosthesis impregnated with gelatine (SealPTFE, Vascutek Terumo, United Kingdom).

### Immobilization technique

The biologically active molecules were covalently immobilized according to the protocol described in subsection 2.3 a)—GLA activation^73^ on the surface of polymeric biomaterials using 5% GLA as a cross-linking agent. After the activation process, the polymeric biomaterials were rinsed with 0.1 M phosphate buffer pH 7 and then the newly formed bonds were reduced with NaBH_4_ (20 μL of 2 mg/mL NaBH_4_ dissolved in distilled water). After the reduction process, the biomaterials were rinsed again with 0.1 M phosphate buffer pH 7 and then the solutions of the molecules at the optimal concentration were added (30 µg/mL of CecA, 40 µg/mL of Pur). Pieces of the prostheses immersed in the inhibitor solutions were shaken for 3 h at room temperature and then kept at + 4 °C for approximately 12 h for stabilisation of the modified surface.

### Kinetics of the release of biologically active molecules

Fragments of modified biomaterials (with dimensions 1 × 3 cm) were placed in sterile 50 mL flasks and filled with 20 mL of sterile phosphate-buffered saline (PBS) at pH 7.4. The flasks were placed on a laboratory shaker and incubated for 30 days (temperature 37 °C, 100 RPM). To determine the amount of released biologically active substances at specified time intervals, a 1 mL sample was taken, and the flasks were replenished with a fresh portion of PBS buffer to maintain a constant volume of 20 mL.

The analysis of the Pur concentration was performed using the HPLC technique on an Agilent Technology 1260 Infinity apparatus equipped with a DAD detector. The separation was performed by reverse phase HPLC on a Phenomenex Luna C18(2) column (4.6 × 150 × 5 µm, 100 Å) with isocratic elution (acetonitrile—eluent A, 50 mM formate buffer—eluent B). The pH of eluent B was adjusted to 4.1 with 1 M NaOH. The ratio of eluent A–B was 30:70 and the elution rate was set to 1 mL/min. During the separation, the column was thermostatted and the temperature was kept constant at 30 °C, the samples were dispensed using an autosampler in a volume of 1 µL, and detection was carried out at 280 nm.

The concentration of CecA in the tested samples was estimated by measuring the intensity of fluorescence, which was carried out on a Tecan SPARK plate reader. The technique of antimicrobial peptide derivatization using *o-*phthalaldehyde (OPA) was used for quantitative analysis. The assay was performed in 96-well plates for fluorescence measurements. 100 µL of the derivatizing agent (OPA working solution—10 mg of OPA was dissolved in a mixture of 200 µL of absolute ethanol (99.6%), 2 µL of β-2-mercaptoethanol, and 20 mL of carbonate buffer pH 10.5) was dosed to 100 µL of the tested sample and mixed, and the absorbance was read after 10 s (excitation wavelength 340 nm, emission wavelength 455 nm).

### Screening of MIC and MBC values

The antimicrobial activity of CecA and Pur was screened with the broth microdilution method with determination of the minimum inhibitory concentration (MIC) and the minimum bactericidal concentration (MBC) on reference strains *E. coli*, *P. aeruginosa*, *S. aureus*, *S. epidermidis,* and *C. albicans* in the concentration range of 1–500 µg/mL. A bacterial suspension with a density of 0.5 McFarland was prepared in sterile distilled water from a fresh 24 h culture on a solid medium, and then diluted in liquid MHB medium to a final density of 10^2^ cfu/mL. Dilutions of CecA and Pur were prepared in a 96-well plate, with the final volume per well of 300 µL. The sterility of the liquid medium and the viability of the tested strains were checked in parallel. The plate was incubated for 24 h at 37 °C. After the incubation, the absorbance of the wells was read using a microplate reader (Tecan SPARK) at 600 nm for MIC determination. For MBC determination, 10 µL of the contents from each well were plated on solid media and incubated for 16–20 h in the conditions described above. The experiment was carried out in three independent replications.

### Reduction of *Staphylococcus epidermidis* growth in suspension cultures after contact with modified biomaterials

The antibacterial activity of the polymeric biomaterials modified with CecA and Pur was assessed in *S. epidermidis* cultures with an *inoculum* density of 10^2^ cfu/mL. Bacterial cultures were carried out on MHB medium in 24-well polystyrene plates at 37 °C with continuous shaking (130 rpm). Each well in the plate contained 950 μL of the culture medium, 50 μL of the native molecule (CecA or Pur) solution or a piece of the modified biomaterial (5 mm × 5 mm), and 10 μL of the *inoculum*. After the 18 h incubation, OD was spectrophotometrically measured at 600 nm on a Tecan SPARK microplate reader. In parallel to the experiment, the sterility of the liquid medium and the viability of the tested strain were checked. The comparison of the culture density data to the control growth (bacterial cells cultured without the addition of the native molecule or the modified biomaterial) was accompanied by calculation of the reduction of living microorganisms [%]. All experiments were carried out in triplicate.

### Antibiofilm properties

The in vitro evaluation of the biofilm production by reference microorganisms (*E. coli, P. aeruginosa, S. aureus, S. epidermidis*, and *C. albicans*) on the surface of modified and unmodified biomaterials was performed in sterile 6-well plates. Each well contained 5 mL of the microorganism suspension in TSB (bacteria) or YMB (yeasts) media with a density of approximately 10^3^ cfu/mL. The samples were incubated for 72 h at 37 °C and 130 rpm, and the TSB medium was replaced with fresh ones every 24 h. After incubation, the prosthesis samples were washed with sterile PBS buffer pH 7.4 and placed on sterile plates in 5 mL of fresh TSB medium. Next, 1% 2,3,5-triphenyltetrazolium chloride (TTC) solution was added and the formation of red formazan was observed. The results of the experiments were documented in photographs (Supplementary materials). SEM micrographs were taken for preparations that showed the greatest potential to inhibit the growth of pathogenic microorganisms and biofilm production.

### SEM microphotography

The fixation process consisted of several steps: washing the biomaterials with 0.1 M PBS pH 7.4, fixation in a 4% GLA solution, second washing with 0.1 M PBS, and dehydration with 25–100% EtOH solutions. Then, the samples were placed on aluminium tables and sputtered with a gold layer in the EMITECH K550X cathode sputtering machine. The surface structure of the denture fragments was observed using a TESCAN VEGA 3 LMU scanning electron microscope.

### MIC and MBC analysis for clinical strains of gram-negative bacteria

The antimicrobial activity of CecA was tested using the broth microdilution method, with determination of the MIC and MBC values in double dilutions of the substance in the range of 0.25–16 µg/mL. CecA activity was tested against *Escherichia coli* reference (ATCC 25922) and 20 clinical strains and against *Pseudomonas aeruginosa* reference (ATCC 27853) and 20 clinical strains; all clinical strains were isolated from skin and soft tissue infections.

MHB solid or liquid media were used in the experiment. A bacterial suspension with a density of 0.5 McFarland, corresponding to approx. 1.5 × 10^8^ cfu/mL, was prepared in a sterile NaCl solution (0.85%) from a fresh 24 h culture on a solid medium and then diluted in liquid medium to a final density of 10^2^ cfu/mL. The dilutions of CecA were prepared in a 96-well plate (Nunc, Thermo Fisher Scientific, USA) with a final volume of 100 µL. In parallel to the experiment, the sterility of the liquid medium and the viability of the tested strains were checked. The plate was incubated for 24 h at 37 °C in an aerobic atmosphere. After incubation, the absorbance of the wells was read using a microplate reader (BioTek ELX 800, USA) at 600 nm for MIC determination. For MBC determination, 5 µL of the contents from each well were plated on solid media and incubated for 16–20 h in the conditions described above. The experiment was carried out in three independent replications.

### Reduction of the growth of clinical gram-negative bacteria in suspension cultures after contact with modified biomaterials

Pieces (approx. 7 × 7 mm) of an unmodified or CecA-modified PPmesh were tested using *E. coli* and *P. aeruginosa* reference strains as well as clinical strains of microorganisms for which the minimum inhibitory concentration of CecA differed from the values obtained with the method of microdilution in the broth for the reference strains (eight and seven clinical strains of *E. coli* and *P. aeruginosa*, respectively). Suspension cultures were grown in 48-well plates (Falcon, Becton Dickinson, USA) using MHB solid or liquid media. A bacterial suspension with a density of 0.5 McFarland (approx. 1.5 × 10^8^ cfu/mL) was prepared from a fresh 24 h culture on a solid medium and then diluted 100-fold to the final density of 10^6^ cfu/mL. Before starting the experiment, the pieces of PPmesh (without CecA) were exposed to UV radiation on both sides (2 × 15 min).

For each of the tested strains, two wells were inoculated with a suspension with a density of 10^6^ cfu/mL in a volume of 1 mL, and then a CecA-modified PPmesh was added to one of the wells. The exact number of bacterial cells in the original suspension was determined by making decimal dilutions and plating 100 µL on a solid medium in duplicate. To exclude the influence of the surgical mesh itself on microbial growth, its fragments were incubated with the reference strains of *E. coli* and *P. aeruginosa*. Additionally, sterile controls of the liquid medium and the unmodified and CecA-modified PPmesh were tested. The plate was incubated for 24 h at 37 °C in an aerobic atmosphere. Then, to determine the number of bacterial cells in the incubation wells, decimal dilutions were made, and 100 µL was plated onto solid supports with the duplicate surface plating technique. The experiment was carried out in three independent replications. To quantify the level of microbial reduction, the results are presented as the mean on a log 10 scale (log_10_ cfu/mL).

### Cell cultures

The studies were performed on human colon normal epithelial cells CCD 841 CoTr (ATCC CRL-1807) cultured in an RPMI 1640:DMEM media mixture (1:1 v/v) with the addition of 10% v/v FBS (Corning, US) and antibiotics (100 U/mL penicillin, 100 μg/mL streptomycin) (Sigma) and incubated at 34 °C in humidified atmosphere with 5% CO_2_ flow. For the analyses, the cells were detached, counted, and diluted to an appropriate density.

Three variants of cultures were used in the in vitro proliferation and cytotoxicity experiments: (1) screening—the classical 2D culture of the cells poured onto a 96-well plate was carried out; (2) direct tests—the cells were cultured on the PPmesh prostheses, forming 3D cultures; (3) indirect tests—2D cultures of the cells poured onto 96-well plates were incubated with PPmesh prosthesis extracts (described further).

#### Cell growth on prostheses

The PPmesh prostheses were incubated in complete culture medium (with 10% FBS and antibiotics) for 24 h. Afterwards, they were transferred to the new wells of the 48-well plate and attached to the bottoms of the wells with silicone grease, and the cell suspension was poured (5 × 10^5^ cells/mL, 500 µL/well). After 24 h, prostheses coated with the cells were transferred to new plate wells and attached to their bottoms. From now on, the cultures were grown for 1 or 4 days.

#### Preparation of extracts

The PPmesh prostheses were incubated in the complete culture medium (volume equal to 1 mL for 0.1 g of the material, according to the ISO norm 10993–12) for 24 h with aspiration. Afterwards, the media (from now on called extracts) were collected and frozen at − 80 °C.

The extracts were prepared from all prosthesis variants: control (unmodified, commercially available PPmesh prosthesis), CecA-PPmesh (prosthesis coated with cecropin A), and Pur-PPmesh (prosthesis coated with puromycin). An additional control, referred to as “medium”, was prepared—the culture medium was treated in the same manner as the other extracts, but without the presence of the prosthesis. The variant served as an additional control.

### Prosthesis cytotoxicity and cell proliferation

**Figure 5:**
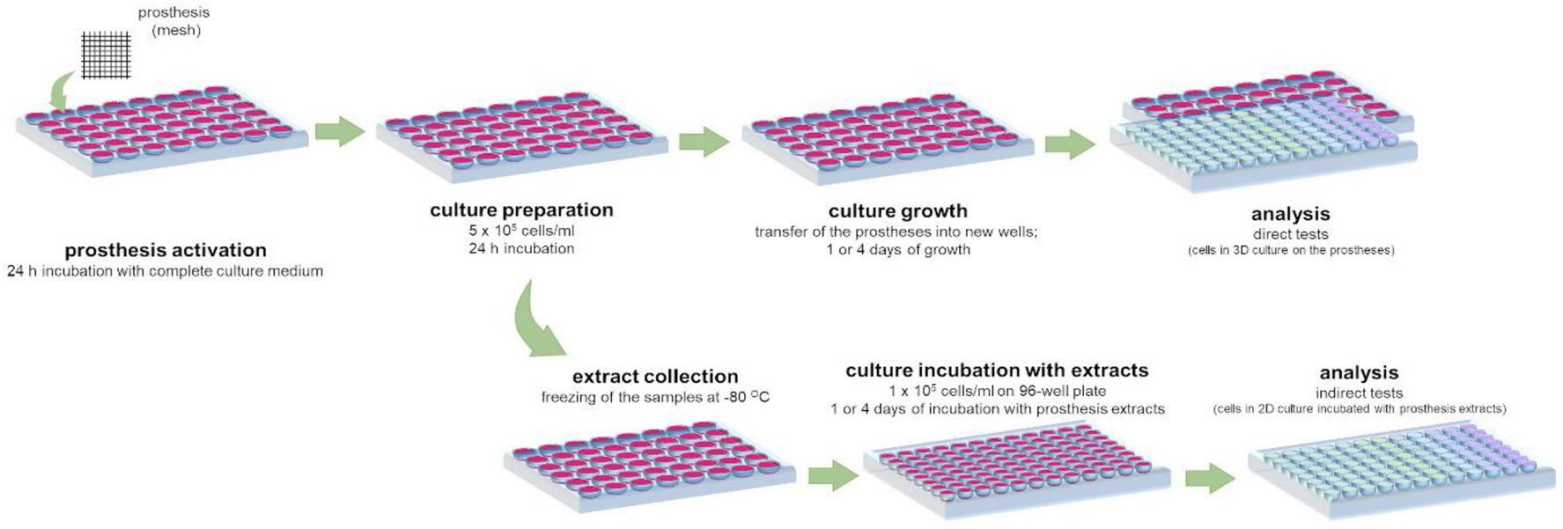
Procedure of culture preparation for further analyses (direct and indirect tests). For the direct tests, the PPmesh prostheses were incubated for 24 h in the complete medium. Afterwards, the cells were seeded (500 of 5 × 10^5^ cells/mL) and incubated for another 24 h. Then, the prostheses were transferred to the new wells and incubated for either 1 or 4 days. For the indirect tests, prosthesis extracts were prepared through incubation of the prostheses in a complete culture medium for 24 h (at a volume equal to 1 mL per 0.1 g of the prosthesis). The extracts were frozen and thawed prior to analysis; cells cultured in 96-well plates were incubated with the extracts for either 1 or 4 days. In both the direct and indirect tests, further analyses were performed after 1 or 4 days of incubation using the cells or post-culture medium, depending on the assay.

MTT and LDH tests were performed to evaluate cell proliferation and cytotoxicity towards the cells, respectively^74^. First, screening analysis on classic 2D cultures in 96-well plates (1 × 10^4^ cells/mL, 100 µL/well) was performed using soluble studied compounds. CecA and Pur were administered at a concentration range of 10–50 µg/mL in 100 µL (compared to the concentration of the compound on the prostheses of approx. 25 µg/mL). The cultures were incubated for 1 day (for cytotoxicity evaluation with the LDH assay) or 3 days (for proliferation evaluation with the MTT method). Next, the direct and indirect tests with the prostheses were performed. The direct tests were carried out using prostheses coated with the cells (3D cultures), while the indirect tests involved the cells in the classic 2D culture incubated with the prosthesis extracts (Fig. 5).

#### Effect of prostheses on cell proliferation

After the incubation of the cells with Pur and CecA (screening) on the prostheses (direct test) or with prosthesis extracts (indirect test), the MTT (Sigma) solution was added to each well, including the blank (culture medium only with no cells) to the final concentration of 1 mg/mL (at the volume of 25 µL for the screening and indirect tests or 125 µL for the direct tests). The plates were then incubated for 3 h at 34 °C. Subsequently, the SDS (Sigma) solution was added to each well (100 µL or 500 µL), and the plates were incubated for 24 h at 34 °C. The absorbance was read using a microplate reader (BioTek) at 570 nm.

#### Prosthesis toxicity towards cells

Cell cultures were prepared as described above; however, additional variants were prepared for evaluation of maximum and spontaneous LDH release according to the manufacturer’s guide (Thermo Fisher cat. C20300). After the incubation, the procedure was carried out according to the instruction—the maximum, spontaneous, and treated samples were prepared through addition of lysis buffer, distilled water, or culture medium, respectively, and incubated for 45 min at 34 °C. All the samples were then poured onto 96-well plates (50 µL of each sample) in triplicates and the reaction mixture was added. After 30-min incubation at room temperature, 50 µL of a stop solution was added and the plates were read at 490 and 680 nm. The cytotoxicity was calculated with the formula:

$$\%cytotoxicity=\left[\frac{treated \;LDH \;activity-spontaneous \;LDH \;activity}{maximum \;LDH \;activity-spontaneous \;LDH \;activity}\right]\times 100\%$$

### Evaluation of the inflammation process

#### Griess method

Nitric oxide (NO) is secreted by cells in various circumstances. One of them is the occurrence or acceleration of the inflammatory process. Therefore, NO may be a factor used in the evaluation of the inflammation state in cells. The NO level was assessed using Griess method, which is based on measurements of the concentration of NO_x_ based on NO_2_ and NO_3_ stable forms^75^.

The 3D cultures were prepared as described above. After the transfer of the cell-coated prostheses into new wells, the cultures were grown for 1 or 4 days. Two experimental variants were prepared: LPS(−) and LPS(+). The LPS(−) variants were cultured without LPS pre-incubation and were carried out as described above. The LPS(+) variants were treated with 10 µg/mL of *E. coli* LPS (Sigma) for 2 h. The pre-incubation was carried out after the transfer of the cell-coated prostheses to new wells. After the pre-incubation, the cells were incubated for 1 or 4 days. The further procedures were performed identically for both variants: after 1 or 4 days, 50 µl of the medium were transferred into the 96-well plate. Subsequently, 50 µl of the Griess reagent (1% sulfanilamide/0.1% *N*-(1-naphthyl)ethylenediamine dihydrochloride (Sigma) in 3% H_3_PO_4_ (Sigma)) were added to each well (samples and standards). NaNO_2_ (Sigma) was used as a standard. After 10-min incubation at RT, the plates were measured spectrophotometrically at 570 nm.

#### ELISA tests

The concentrations of inflammation mediator COX-2 and Th2-type immune response cytokines (IL-4. IL-10, IL-13) were measured using ELISA tests (COX-2—Thermo Scientific, US, IL-4/10/13—Biorbyt, UK). The cultures were carried out as described in the previous section. For the COX-2 measurements, the cells on the prostheses were lysed using Lysis Buffer (Thermo), the concentration of proteins was measured, and the samples were diluted. For the determination of cytokines, media from the 3D cultures were collected. Afterward, the samples were frozen at − 80 °C. Next, they were thawed and mixed gently before analysis. The further steps were performed according to the manufacturer’s guides.

#### Cytokine array

The cells were grown on the prostheses for 4 days (as described earlier); next, the media were collected and pooled from 3 repeats. Subsequently, the samples were frozen at -80 °C. The samples were thawed and mixed gently before analysis. The procedure was carried out according to the manufacturer’s guide (R&D Systems cat. ARY005B); 1 mL of each sample was used. The membranes were visualized with ChemiDoc XRS + (BioRad, US), and densitometric analysis was performed using ImageJ software (NIH, US). The results were calculated compared to the control set to 100% (results from cells cultured on control prosthesis in LPS(−) conditions).

### Statistical analysis

Results from 3 repeats were presented as mean ± SD. Statistical evaluation was performed using GraphPad Prism software. One-way ANOVA with Dunnett’s or Tukey’s test was performed and the results were indicated with “*” (compared to the control – unmodified prosthesis) or, in particular analyses (indirect tests), also with “^#^”, indicating significant differences from the medium variant (cells incubated with the extract prepared without the prosthesis).

### Supplementary Information

Supplementary Tables.

## Data Availability

The datasets generated during and/or analysed during the current study are available from the corresponding author on reasonable request.
