# Supplementary material for: Modified polymeric biomaterials with antimicrobial and immunomodulating properties
Source: Sci Rep. 2024 Apr 5;14:8025. doi: 10.1038/s41598-024-58730-3 (PMC10997598; doi:10.1038/s41598-024-58730-3)
Supplement: Supplementary file 1 — Supplementary Tables. [file 41598_2024_58730_MOESM1_ESM.docx]

**Supplementary materials**

Table S1. Photos of the **PPmesh** – unmodified surgical mesh (PPmesh) and material modified with cecropin C (CecA-PPmesh) or puromycin (Pur-PPmesh) incubated with various microorganisms after the completed TTC staining - visualization of the resulting biofilm

| **Microorganism** | **Pur-PPmesh** | **CecA-PPmesh** | **Unmodified PPmesh** |
| --- | --- | --- | --- |
| *Escherichia  coli*  ATCC 25922 | 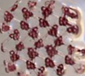 | 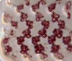 | 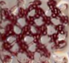 |
| *Staphylococcus aureus*  ATCC 25923 | 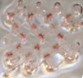 | 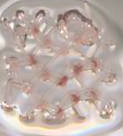 | 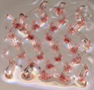 |
| *Staphylococcus epidermidis*  ATCC 14990 | 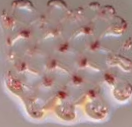 | 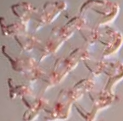 | 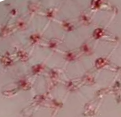 |
| *Pseudomonas aeruginosa*  ATCC 27853 | 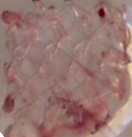 | 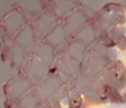 | 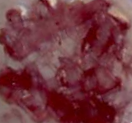 |
| *Candida albicans*  ATCC 10231 | 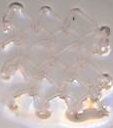 | 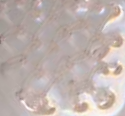 | 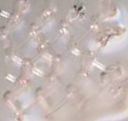 |

Table S2. Photos of the **ePTFE** – unmodified vascular prosthesis (ePTFE) and material modified with cecropin C (CecA-ePTFE) or puromycin (Pur-ePTFE) incubated with various microorganisms after the completed TTC staining - visualization of the resulting biofilm

| **Microorganism** | **Pur-ePTFE** | **CecA-ePTFE** | **Unmodified ePTFE** |
| --- | --- | --- | --- |
| *Escherichia  coli*  ATCC 25922 | 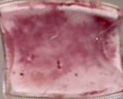 | 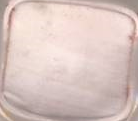 | 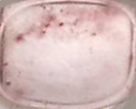 |
| *Staphylococcus aureus*  ATCC 25923 | 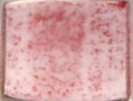 | 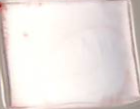 | 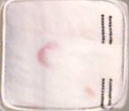 |
| *Staphylococcus epidermidis*  ATCC 14990 | 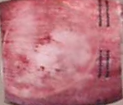 | 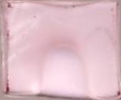 | 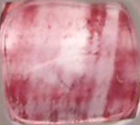 |
| *Pseudomonas aeruginosa*  ATCC 27853 | 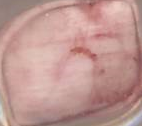 | 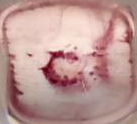 | 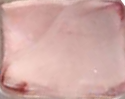 |
| *Candida albicans*  ATCC 10231 | 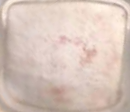 | 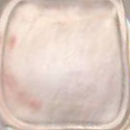 | 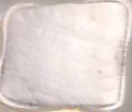 |
